# Validation of a Portable Fluorescence Spectroscopy System to Monitor Heat Damage in Industrially Processed Milk

**DOI:** 10.3390/foods13050780

**Published:** 2024-03-02

**Authors:** Ulises Alvarado, Anna Zamora, Oscar Arango, Jordi Saldo, Manuel Castillo

**Affiliations:** 1 Centre de Innovació, Recerca i Transferència en Tecnologia dels Aliments (CIRTTA), Animal and Food Science Department, Facultat de Veterinària, Universitat Autònoma de Barcelona, 08193 Bellaterra, Spain; ualvarado@unap.edu.pe (U.A.); jordi.saldo@uab.cat (J.S.); manuel.castillo@uab.cat (M.C.); 2 Escuela Profesional de Ingeniería Agroindustrial, Facultad de Ciencias Agrarias, Universidad Nacional Del Altiplano, Av. Floral 1153, Puno 21001, Peru; 3 Facultad de Ingeniería Agroindustrial, Universidad de Nariño, Ciudad Universitaria Torobajo, Pasto 47154, Nariño, Colombia; oscar.arangob@gmail.com; 4 Centro de Investigación de Alimentos (CIAL), Grupo de Procesamiento de Alimentos, Universidad UTE, Quito 170147, Ecuador

**Keywords:** fluorescent compounds, heat damage, quantification, spectrofluorometer, milk, prediction

## Abstract

Heat treatments play a critical role in ensuring the safety and preservation of milk, but it can affect its nutritional and sensory properties. The present paper proposes the use of a portable system based on fluorescence spectroscopy as an alternative method for the quantification of four thermal damage markers at once (hydroxymethylfurfural, sulfhydryl groups, ascorbic acid, and riboflavin). The obtained prediction models using autofluorescent compounds (tryptophan, dityrosine, Maillard compounds, and riboflavin), validated with skimmed milk processed under several industrial conditions, granted the development of a portable and/or online system, allowing for the real-time monitoring of thermal damage and control of the heat treatment process. The results of this study will certainly contribute to the development of new process analytical technologies for the dairy industry, enabling quality control and adjustment of the manufacturing process to ensure safe and high-quality products.

## 1. Introduction

Heat treatment is a crucial step in the dairy industry to sanitize and preserve milk by inactivating enzymes and destroying microorganisms before consumption. However, the intensity of these heat treatments can also alter the nutritional value and organoleptic properties of the final product, such as the color, smell, and flavor. To monitor and control the extent of thermal damage, the dairy industry has identified different chemical markers, which are classified into two types. Type 1 markers are related to the degradation, denaturation, and inactivation of thermolabile components, including whey proteins, enzymes, and vitamins. On the other hand, type 2 markers are associated with the formation of new substances, such as lactulose and products of the Maillard reaction [1]. Therefore, to ensure the safety and quality of dairy products, it is essential to balance the benefits of heat treatment against its potential negative effects on the nutritional value and sensory characteristics of the final product.

In the present investigation, we will focus on the study of four thermal damage markers. Hydroxymethylfurfural (HMF) is a type 2 marker, being an intermediate of the Maillard reaction. It is generally used in the dairy industry as a marker of the effect of heat treatment on milk quality [2]. Sulfhydryl groups (-SH), ascorbic acid (AA), and riboflavin (Rbf) are three type 1 markers. β-Lactoglobulin is a milk protein that contains the highest concentration of sulfur-containing amino acids, which are normally inaccessible in the inner part of the dimer. Because of heat treatment, the protein is denatured, unfolding and exposing the -SH groups. This allows for the measurement of -SH groups to be used as an index of heating in milk [3]. AA is an important and essential nutrient for humans, and although milk is unfortunately not a good source of AA, its presence is of interest due to its potential use as an indicator of thermal damage or process quality [4]. Rbf is also essential for humans and milk is the main source. Several authors agree that heat treatment has only insignificant effects on the concentration of Rbf since it is one of the least heat-labile water-soluble vitamins; however, this vitamin could be an alternative to evaluate the thermal damage of skimmed milk, since in whole milk the Rbf is protected by fat globules that appear to have a blocking or suppressive effect on the thermal load [5].

Traditionally, chemical markers are quantified by evaluating their concentrations using conventional analytical methods. For example, the concentrations of AA, Rbf, and HMF are normally quantified by high-performance liquid chromatography (HPLC) methods [6,7,8,9], and for the quantification of the -SH groups, the Ellman method is usually used [10,11]. These analytical methods do not allow for the continuous and easy monitoring of the process because they are expensive methodologies, require sophisticated equipment and personnel with appropriate professional qualifications, and in most cases require sample extraction or pre-treatment steps, increasing the time needed for analysis. Consequently, they are not suitable for online quality control, which implies a risk of obtaining unsafe or low-quality food during production, and they also do not allow for the the correction of the technological process to adjust the resulting imbalance in real time due to the long period until the results are available. Consequently, there is a need to develop process analytical technologies to measure the quality and safety of food during manufacturing [12].

Fortunately, at present, front-face fluorescence (FFF) spectroscopy is an analytical tool that has great potential for the evaluation of compositional and nutritional properties of dairy products. Studies have recently been carried out to predict changes in analytes such as lactulose [13], furosin [14], retinol [15], and β-lactoglobulin [16] during the heat treatment of milk and also to determine Rbf in commercial kinds of milk [17]. Most of these studies were performed using a benchtop spectrofluorometer in steady-state measurements and showed promising results overcoming many of the drawbacks of conventional methods. However, it is not possible to couple equipment with these characteristics to online quality control systems because the measurements are not carried out in situ. Currently, expectations are being raised by the use of portable fluorescence spectroscopy systems, made up of a mini spectrophotometer and a light source based on LEDs connected by fiber optics to the sample and which are all controlled from a laptop. This innovative equipment is useful for measuring on-site or online, which makes them ideal for real-time monitoring, offering an important advantage in the food industry.

Our research group has carried out studies showing that FFF is a viable alternative to determine the effect of the heat treatment of milk on the content of various compounds such as lactulose [13], retinol [15], and Rbf [17]. Alvarado et al. [18] evaluated the potential of FFF spectroscopy to predict the AA and Rbf concentration in milk after thermal processing, developing models with coefficients of variation smaller than 3.57% for Rbf and ranging from 4.24 to 14.25% for AA.

The mentioned studies were carried out using a desktop spectrofluorometer, which, due to its characteristics, does not allow for the online monitoring of the effects of heat treatments on the nutritional quality of milk. Thus, the ultimate goal of the research was to develop a viable technology that would allow the dairy industry to quantify the effects of heat treatment on milk quickly and non-destructively by acquiring online measurements and enabling real-time monitoring of the process. And the objective of the present work was to validate the potential application of this portable fluorescence spectroscopy system, which allows for the simultaneous quantification of several thermal damage markers in milk processed under industrial conditions such as thermization, High-Temperature Short-Time (HTST) pasteurization, Higher-Heat Shorter-Time (HHST) pasteurization, Ultra-High-Temperature (UHT) continuous sterilization, and conventional sterilization. 

## 2. Materials and Methods

### 2.1. Reception of Milk

The study was carried out with whole raw milk from the Can Badó farm (La Roca del Vallès, Barcelona, Spain). Approximately 150 L was used for each replication, which was previously skimmed (Seital, R.L.S. Model SE 02.0V, Santorso, Italy). The milk was subjected to various heat treatments commonly used in the industry at the Food Technology Plant of the Universitat Autònoma de Barcelona.

### 2.2. Heat Treatments in Industrial Conditions

The heat treatments applied to the skimmed milk were carried out in a tubular pasteurizer (Tetra Therm^®^ Aseptic Pilot, Tetra Pak, Lund, Sweden). These treatments were thermization (65 °C, 15 s), HTST pasteurization (72 °C, 15 s), HHST pasteurization (105 °C, 2 s), and UHT sterilization (138 °C, 4 s). Also, bottle sterilization (120 °C, 20 min) was carried out in an autoclave (model 1000-LC ref. 1024, Calderería Ramón Naves S.L., Barcelona, Spain). After each heat treatment, the samples were maintained at 20 °C to measure the fluorescence. For the determination of the chemical markers (AA, Rbf, HMF, and -SH), the samples were frozen at −30 °C until further analysis.

### 2.3. Quantification of Thermal Damage Markers

#### 2.3.1. Hydroxymethylfurfural

The technique used for the chemical determination of HMF has been defined in a study by Keeney and Bassette [19]. This method determines the total HMF, which includes not only the concentration of free HMF detected in milk that is produced from the Maillard reaction (decomposition of the Amadori product lactulosyl-lysine) but also the HMF formed by lactose degradation via Lobry De Bruyn–Alberda van Ekenstein (LA) transformation, which, in fact, is the major pathway for the formation of HMF in milk [20].

Aliquots of 10 mL of reconstituted milk were used with the addition of 5 mL of 0.3 N oxalic acid (18.9 g of oxalic acid diluted in 1 L of distilled water). The mixture was placed in 20 mL Pyrex glass tubes that were kept, covered, in an oven at 102 °C for 1 h. Subsequently, the tubes were cooled with cold water to room temperature (~21 °C) and 5 mL of 40% trichloroacetic acid (TCA) in distilled water (*w*/*w*) was added and, after mixing, filtered through a Whatman No. 42 filter. After filtration, 4 mL of the permeate was pipetted into a 15 mL Pyrex tube and 1 mL of 0.05 M thiobarbituric acid (TBA) prepared with distilled water was added. After the mixture was homogenized, it was kept for 40 min in a thermostatic bath at 40 °C, after which the sample was cooled to room temperature, as previously indicated.

Finally, the absorbance at 443 nm was read with a spectrophotometer (mod. UV2310, Dinko Instruments, Barcelona, Spain) using a Suprasil^®^ quartz cuvette (mod.104-QS, Hellma GmbH & Co. KG, Müllheim, Germany) against a blank prepared in the same way but substituting milk for water.

To calculate the HMF concentration (µmol·L^−1^), the following equation provided by the abovementioned study was used:

$$\left[HMF\right]\left(µmol\cdot L^{-1}\right)=\left(A_{443nm}-0.055\right)\cdot 87.5$$

#### 2.3.2. Sulfhydryl Groups

For the determination of the total sulfhydryl/thiol (-SH) groups in the milk samples, the rapid determination method proposed by Guingamp et al. [10] was used. A 0.5 mL aliquot of the skimmed milk was collected and 0.5 mL of distilled water (1:1 (*v*/*v*)), 1 mL of 8 M urea buffer (pH 8.5), and 50 µL of DTNB were added. The mixture was homogenized by vortexing and allowed to settle for 5 min at room temperature. Subsequently, 0.5 mL of 0.2 M EDTA (pH 6) and 2 mL of specific clarifying agent for dairy products (Clarificant Reagent^®^ for dairy products, Sigma Aldrich Co., St. Louis, MO, USA), previously tempered in a thermostatic bath at 37 °C, were added to the mixture. After homogenizing the sample in a vortex, it was left in a thermostatic bath at 37 °C for 10 min. Finally, the sample was cooled in ice water to 21 °C and immediately after, the absorbance at 412 nm was measured against a blank (blank 1) containing all reagents except milk. The correct absorbance of the samples was verified by reading the absorbance of blank 2 (without DTNB) against blank 3 (without milk and without DTNB). The concentration of -SH groups was calculated from a calibration curve of L-cysteine. The results were expressed in µmol -SH·g^−1^ of total solids, not including fat.

#### 2.3.3. Riboflavin

Rbf concentration was analyzed using the method described by Albalá-Hurtado et al. [21]. Thawed milk samples (5 g) were accurately weighed into a centrifuge tube, to which 0.5 g trichloroacetic acid was added. The mixture was shaken for 10 s and subsequently centrifuged for 15 min at 2500 rpm (Sigma laboratory centrifuge, 4K-15, SN. 93250, Osterode am Harz, Germany). The supernatant was stored and 0.5 mL of 4% trichloroacetic acid was added to the residual pellets for a second extraction by mixing for 1 min before centrifuging at 2500 rpm for 10 min. The two acid extracts were combined in a 10 mL tube and kept protected from light. Just prior to HPLC injection, the extracts were filtered through 0.22 µm filters.

A 100 mg·L^−1^ stock solution of Rbf in the form of flavin mononucleotide (R9504, Sigma Aldrich, Saint Louis, MO, USA) prepared in 2.4% (*v*/*v*) acetic acid was used to obtain calibration solutions (0.5, 1.0, 2.0, and 4.0 mg·L^−1^), diluting the stock solution in 2.4% aqueous acetic acid. All standard solutions were filtered through a 0.22 µm filter just before HPLC injection.

Chromatography measurements were performed in triplicate using a Dionex P680 HPLC UV-Vis detector (Dionex, Germering, Germany). The analytical column used was Waters Spherisorb ODS 2 C18, packed with 5 μm diameter, 4.6 × 150 mm particles (Tecknokroma, Barcelona, Spain). Chromatographic separation was performed under isocratic conditions in a mobile phase of 5 mM octane sulfonic acid, 0.5% triethylamine, 24% glacial acetic acid, and 15% methanol. The eluent flow rate was 1.0 mL·min^−1^ and the column temperature was 25 °C. The analysis time for each sample was 20 min.

#### 2.3.4. Ascorbic Acid

The same Dionex HPLC, equipped with a Tracer Extraxil ODS 2 C18 analytical column and 0.46 mm × 250 nm and 5 × 5 mm ID diameter particles (Tecknokroma, Barcelona, Spain) was used for the quantification of AA following the method described by Romeu-Nadal et al. [22] with some modifications. Thawed milk samples (1.50 mL) were centrifuged at 12,000 rpm for 15 min (Hettich Universal, Mikro 12–24, Berlin, Germany). A total of 0.4 mL of the supernatants were carefully removed, mixed with 1 mL of 0.55% (*w*/*v*) metaphosphoric acid solution, and manually shaken for 30 s. Supernatants obtained by a second centrifugation at 12,000 rpm for 10 min were filtered through a 0.22 µm filter just before HPLC injection.

Chromatographic separation was performed in triplicate by isocratic elution using a Milli-Q water mobile phase with acetic acid (0.1% *v*/*v*) and methanol in a 95:5 (*v*/*v*) ratio. The eluent flow rate was 0.7 mL·min^−1^, the column temperature was 25 °C, and the analysis time for each sample was 20 min. AA was identified by comparing the retention time of the sample peak at 254 nm. Quantification was performed using a calibration curve of AA standard solutions (1, 5, 10, 20, and 30 ppm) prepared by dissolving AA (Sigma-Aldrich, Madrid, Spain) in 0.55% (*w*/*v*) metaphosphoric acid dissolved in Milli-Q water.

### 2.4. Fluorescence Determination

Fluorescence measurements were performed with a Model QEPro portable spectrophotometer (Ocean Optics, Inc., Dunedin, FL, USA), longpass filters (kits #47-846 and #47-848; Edmund Optics, Ltd., Nether Poppleton, York, UK), two lamps each containing three LEDs with different wavelengths (Ocean Optics, Inc., Dunedin, FL, USA), and a prototype cell which consisted of a 40 mL cylindrical black plastic base with a 2.54 cm diameter hole for inserting the fiber optic probe, which consisted of two parallel optical fibers (Ocean Optics, Inc.). The working conditions of the portable spectrophotometer were as follows: Trp fluorescence spectrum (F_Trp_) was scanned at 290 nm of excitation and in the range of 300 to 450 nm of emission, Maillard compound fluorescence was measured at a wavelength of 325 nm of excitation and between 350 and 500 nm of emission, for dityrosine, the excitation wavelength was 315 nm while the emission ranged from 350 to 500 nm, Rbf was excited at 265, 365, and 455 nm (F_Rbf265_, F_Rbf365_, and F_Rbf455_, respectively) with an emission wavelength range of 470 to 570 nm. Fluorescence spectra were recorded with SpectraSuite^®^ 2.0 software (Ocean Optics, Inc.). All measurements were performed in triplicate.

For the construction of mathematical prediction models using fluorescent markers, the maximum value of fluorescence intensity of each fluorophore was used.

### 2.5. Obtaining Prediction Models Using Fluorescent Compounds

The data were processed and analyzed using the “Statistical Analysis System” (SAS, version 9.2, 2009, SAS institute Inc., Cary, NC, USA). The maximum R^2^ procedure was used to obtain the best one-, two-, and three-parameter models to predict the concentration of thermal damage markers. For calibration, the maximum fluorescence intensity value of the three replicates of each fluorophore was used. The models’ validation was carried out using the leave-one-out cross-validation method, which consists of validating the same calibration data, but leaving a replicate out each time. The cross-validation technique builds confidence to test the predictive potential of mathematical models. Wong [23] indicates that cross validation should be adopted when the number of cases is small, taking full advantage of the number of cases, to obtain as accurate a calculation of estimated values as possible.

The goodness of fit for the models was evaluated with the parameters R^2^ (determination coefficient), SEC (standard error of calibration), SECV (standard error of cross-validation), and CV (coefficient of variation), as described by Williams and Sobering [24].

## 3. Results and Discussion

### 3.1. Analysis of Variance of Thermal Damage Markers

Table 1 shows the results of the heat damage markers evaluated in skimmed milk subjected to different heat treatments used in the industry. Only in the UHT and sterilization treatments, the obtained HMF concentrations were significantly different from each other and with the rest of the treatments. The concentration of this compound was detected in variable amounts ranging from 1.501 to 43.85 µmol·L^−1^. Morales and Jiménez-Pérez [20] also obtained similar values in raw milk, thermized milk (65 °C, 10 s), and pasteurized milk (85 °C, 30 s), with values of 1.0, 1.5, and 2.5 µmol·L^−1^, respectively. Likewise, Morales et al. [25] obtained HMF concentrations of 8.7 and 22 µmol·L^−1^ in UHT (140 °C, 3 s) and sterilized (116 °C, 15 min) milks, respectively. The difference between our results (43.85 µmol·L^−1^) and the latter values is probably due to the application of a higher temperature and time in our treatment. HMF is a low-molecular-weight molecule formed as an intermediate of the Maillard reaction or by lactose degradation [26] and it has been confirmed that the formation of HMF increases drastically as temperatures and durations of heat treatments increase, as was mentioned by Capuano and Fogliano [27]. On the other hand, amino acid residues such as lysine could easily react with lactose to form HMF in a higher proportion [20].

According to the results and bibliographical references, HMF is useful for distinguishing milk subjected to high temperatures, such as in UHT and bottle sterilization, from that with more moderate treatments. The results corroborate what was mentioned by Pellegrino et al. [28], who stated that HMF is a well-known indicator for monitoring quality changes in food during severe thermal processes. In addition, there are some food hazard concerns related to HMF, because it has observed genotoxic, cytotoxic, and tumor effects [29].

The concentrations of sulfhydryl groups (-SH) can be seen in Table 1. After the heat treatment, the protein unfolds as a product of denaturation, exposing the active -SH groups and generating disulfide bonds, which causes a decrease in the total -SH in milk.

The control and the other treatments were significantly different from each other, except between the thermized and HTST milks. In the raw skimmed milk, the concentration of total -SH was ~1.38 µmol·g^−1^. This result is within the range of 0.72–2.21 µmol·g^−1^ obtained by Guingamp et al. [10,30]. The decrease in -SH in the HHST pasteurized milk was ~36%, a value close to that observed by Guingamp et al. [10] who obtained a 32% reduction in pasteurized milk. The UHT treatment caused a decrease in -SH of up to ~53%, a value lower than the 72% found by Cosio et al. [31] in commercial UHT milk. The bottled sterilized milk presented a ~91% decrease in -SH, a value greater than the 73% reported by Taylor and Richardson [32] in sterilized skimmed milk at 130 °C for 30 min. Guingamp et al. [10] and Cosio et al. [31] found -SH losses of 83% and 88%, respectively, in sterilized commercial milk. The use of different autoclave systems may be responsible for the variations found. In our case, the static batch autoclave caused an intense effect on the samples.

The AA content in milk varies from 8.5 to 27.5 mg·L^−1^ [33], being low compared to other foods such as fruit juice and some vegetables. In addition, it is considered the least stable vitamin, and can undergo oxidation, favored by the presence of metals or exposure to light and, clearly, by high temperatures [33].

The AA value in the raw skimmed milk was 21.77 mg·L^−1^ (Table 1). Some authors have reported seasonal differences in the AA concentration, being higher in winter [34,35]. In addition to the season of the year, the breed of the cow and the stage of lactation appear to be important factors causing variation in the AA content of fresh milk [36].

The percentage loss of AA in milk subjected to HHST pasteurization reached up to ~24%, a value similar to that mentioned by Fox et al. [34] and Walstra et al. [33] of up to 25%. The UHT treatment caused losses up to ~29%, a value that agrees with that reported by Walstra and Jenness [37]. With bottle sterilization, the AA concentration decreased to ~66%, similar to reports by Ryley and Kajda [38] and Walstra and Jenness [37], who indicated losses greater than 50%. The thermal sensitivity of AA means that this vitamin can be a good indicator to identify different thermal treatments applied to milk.

Milk and dairy products are an important source of Rbf. This vitamin is considered heat stable, and labile to light and oxygen [39]. The Rbf concentration in raw skimmed milk was 1.33 mg·L^−1^ (Table 1). This result agrees with that obtained by Güneşer and Karagül-Yüceer [40] and Asadullah et al. [41], who found values between 0.64 and 1.15 mg·L^−1^ of Rbf. Other authors have found Rbf concentrations greater than 1.7 mg·L^−1^ [33,39]. These differentiations depend on diet, breed, and season, among other factors [34].

The Rbf losses due to the thermization, HTST, HHST, UHT, and bottle sterilization treatments were ~0.7, ~3, ~5, ~7, and ~21%, respectively. Meha [42] found a loss of 1% at heat treatment at 73 °C for 15 s, which is very similar to our result. Sunaric et al. [43] found a loss of 7.1% in commercial UHT skimmed milk, similarly to the results of our UHT treatment. Nohr et al. [39] found a loss of ~18% in bottle-sterilized skimmed milk, a value that is slightly lower than our result, but our results, like those of Nohr et al. [39], are higher than the data obtained by Veisseyre [44], which shows losses < 10%.

Only the bottle-sterilized milk presented a significantly lower Rbf concentration compared to the other samples, which coincides with what was observed by Badui [45] and Fox et al. [34]. These results confirm the stability of Rbf under heat, and not being significantly affected by pasteurization and UHT processes. However, the bottle-sterilized milk had a significantly lower Rbf concentration compared to the rest of the samples, meaning that Rbf alone could only allow for the identification of intense heat treatments of milk.

### 3.2. Prediction Models for Indicators of Thermal Damage in Milk

Using the SAS maximum R^2^ procedure, the best one-, two-, and three-parameter models for the prediction of thermal damage indicators were obtained. Table 2 shows the goodness-of-fit indicators (R^2^, SEC, SECV, and CV) both for the calibration and for the cross-validation of the four thermal damage markers, as well as the coefficients of the mathematical prediction models.

In the algorithms for the prediction of the HMF concentration, the main predictor was F_Trp_, probably due to its strong inverse relationship with [HMF] (r = −0.96; *p* < 0.0001). The clear relationship between this compound and F_Trp_ was also observed in a similar experiment using benchtop fluorescence equipment [46]. In general, a similarity between the CVs of the calibration and the cross-validation of the prediction algorithms can be seen. HMF was the compound that presented with the highest CV, because in the sterilization treatment, the HMF concentration was much higher (43.85 µmol·L^−1^) compared to the other treatments.

The models for predicting the HMF concentration in milk after heat treatment had CV values lower than 45%. Alvarado [46] found values of HMF higher than 64% using a benchtop spectrofluorometer. Model I in Table 2 with a lower SEP (SECV) value (1.51 µmol·L^−1^) could be selected as the best to predict the HMF concentration, because it only has one predictor, which facilitates the calibration.

The main predictor of estimating [-SH] was λ_Ftrp_, which presented a correlation with [-SH] with r = −0.94 (*p* < 0.0001) (data not included). In our previous study, this predictor only had a correlation with r = −0.41 in relation to -SH degradation. The [-SH] prediction models obtained using the portable spectrofluorometer gave CV values < 17%, while those generated with a benchtop spectrofluorometer gave CV values greater than 34% [46].

In the case of the mathematical models for the estimation of AA, it is observed that the main predictor is λ_Ftrp_. Said parameter presented an inverse correlation with [AA] (r = −0.92, *p* < 0.0001). In a recent study, Alvarado et al. [18] obtained models to estimate the AA concentration with a lower goodness of fit (R^2^ = 0.75, SEP = 0.97 mg·L^−1^) using a benchtop spectrophotometer. However, in the present study, more reliable mathematical models were obtained, since the goodness of fit was > 0.80 and the prediction uncertainty was <0.94. The best model turned out to be the one with only one predictor, which could be a fast and easy alternative to predict the AA concentration in skimmed milk after its heat treatment.

For the estimation of Rbf, the F_Rbf370_ predictor was the most important. This predictor was inversely related to the Rbf concentration, with r = −0.80 (*p* < 0.0001). The cross validation of the Rbf prediction models gave values of SECV < 0.040 mg·L^−1^ and R^2^ < 0.55; these results were slightly lower compared to the models obtained by Alvarado et al. [17] and Alvarado et al. [18], both using a benchtop spectrofluorometer. These models should be improved by incorporating more calibration observations prior to their industrial application, given their low R^2^.

## 4. Conclusions

This study pioneers the application of a portable spectrofluorometer for the rapid, label-free quantification of milk thermal damage by monitoring fluorescent changes in four key chemical markers: sulfhydryl and ascorbic acid degradation, hydroxymethylfurfural formation, and riboflavin stability. The intensity of thermal processing directly correlated with changes in the first three markers, highlighting their sensitivity as heat damage indicators. Notably, riboflavin exhibited greater stability, only showing significant alterations under high-temperature sterilization. Prediction models were successfully developed to estimate these chemical alterations based solely on fluorescence spectroscopy data, eliminating the need for sample manipulation and chemical reagents. This innovative approach presents a potentially transformative tool for the dairy industry, offering the real-time, simultaneous monitoring of multiple heat damage markers through online process control integration. Additionally, the portability and label-free nature of the method offers significant advantages in terms of convenience and sustainability, paving the way for more efficient and environmentally friendly quality control measures in milk processing.

## Figures and Tables

**Table 1:** Average concentration of thermal damage markers evaluated in skimmed milk produced under industrial conditions.

	HMF (µmol·L^−1^)	-SH (µmol·g^−1^)	AA (mg·L^−1^)	Rbf (mg·L^−1^)
Raw milk	1.50 ^a^	1.38 ^a^	21.77 ^a^	1.33 ^a^
Thermization	1.52 ^a^	1.26 ^b^	20.27 ^b^	1.32 ^a^
HTST pasteurization	1.65 ^a^	1.21 ^b^	18.33 ^c^	1.30 ^a^
HHST pasteurization	2.84 ^a^	0.88 ^c^	16.58 ^d^	1.27 ^a^
UHT sterilization	9.57 ^b^	0.65 ^d^	15.48 ^d^	1.24 ^a^
Bottle sterilization	43.85 ^c^	0.12 ^e^	7.39 ^e^	1.04 ^b^

N = 18; HMF: hydroxymethylfurfural; -SH: sulfhydryl groups; AA: ascorbic acid; Rbf: riboflavin. Different letters in the same column indicate statistically significant differences (*p* < 0.05). Raw milk: control; thermization: 65 °C, 15 s; HTST pasteurization: 72 °C, 15 s; HHST pasteurization (105 °C, 2 s); UHT sterilization: 138 °C, 4 s; bottle sterilization: 120 °C, 20 min.

**Table 2:** Coefficients of the prediction models for markers of heat damage in milk with different industrial treatments.

Mathematical Models	Coefficients of Regression	Calibration			Validation		
		R^2^	SEC	CV	R^2^	SECV	CV
I. [HMF] = β_0_ + β_1_F_Trp_	**β_0_** = 57.06 * β_1_ = −0.54 *	0.92	4.55	44.80	0.87	1.51	44.67
II. [HMF] = β_0_ + β_1_F_Trp_ + β_2_F_Rbf370_^2^	**β_0_** = 93.27 * β_1_ = −0.68 * β_2_ = −1.59 × 10^−9^	0.93	4.37	43.02	0.86	1.85	42.73
III. [HMF] = β_0_ + β_1_F_Trp_ + β_2_F_Rbf370_^2^ + β_3_F_Rbf450_^−1^	**β_0_** = 146.9 * β_1_ = −0.72 * β_2_ = −2.50 × 10^−9^ * β_3_ = 226,061	0.94	4.14	40.70	0.87	2.15	40.42
IV. [-SH] = β_0_ + β_1_λ_Ftrp_^−1^	**β_0_** = −32.85 * β_1_ = 11,609 *	0.89	0.15	16.74	0.86	0.05	16.75
V. [-SH] = β_0_ + β_1_λ_Ftrp_^−1^ + β_2_F_Rbf450_^2^	**β_0_** = −28.52 * β_1_ = 10,369 * β_2_ = −1.82 × 10^−8^ *	0.92	0.14	14.96	0.89	0.06	14.95
VI. [-SH] = β_0_ + β_1_λ_Ftrp_^−1^ + β_2_F_Rbf450_^2^ + β_3_λ_Ftrp_	**β_0_** = −31.8 **β_1_** = 10,937 **β_2_** = −1.82 × 10^−8^ * β_3_ = 4.7 × 10^−3^	0.92	0.04	15.29	0.88	0.07	15.52
VII. [AA] = β_0_ + β_1_λ_Ftrp_^2^	**β_0_** = 194.32 * β_1_ = −0.001 *	0.84	2.03	12.22	0.81	0.70	12.22
VIII. [AA] = β_0_ + β_1_λ_Ftrp_^2^ + β_2_F_Rbf370_^−1^	**β_0_** = 230.94 * β_1_ = −0.002 * β_2_ = −1,319,764	0.85	2.00	12.04	0.80	0.88	12.04
IX. [AA] = β_0_ + β_1_λ_Ftrp_^2^ + β_2_F_Rbf370_^−1^ + β_3_F_Trp_^−1^	**β_0_** = 190.71 * β_1_ = −0.001 * β_2_ = −2,555,009 * β_3_ = −211.42 *	0.90	1.76	10.56	0.80	0.94	10.52
X. [Rbf] = β_0_ + β_1_F_Rbf370_^2^	**β_0_** = 1.89 * β_1_ = −4.19 × 10^−11^ *	0.65	0.08	6.73	0.54	0.028	6.70
XI. [Rbf] = β_0_ + β_1_F_Rbf370_^2^+ β_2_λ_Ftrp_^−1^	**β_0_** = −2.27 * β_1_ = −2.71 × 10^−11^ * β_2_ = 1352.5	0.69	0.08	6.48	0.53	0.035	6.46
XII. [Rbf] = β_0_ + β_1_F_Rbf370_^2^ + β_2_λ_Ftrp_^−1^ + β_3_λ_Ftrp_	**β_0_** = −475.71 * β_1_ = −4.21 × 10^−11^ * β_2_ = 82,747 **β_3_** = 0.69	0.75	0.07	6.02	0.53	0.039	5.98

N = 18; R^2^: coefficient of determination; SEC: standard error of calibration (same units as predicted variables); SECV: standard error of cross-validation (same units as predicted variables); CV (%): coefficient of variation; β_0_, β_1_, β_2_, and β_3_: regression coefficient; * *p* < 0.05; [HMF]: concentration of hydroxymethylfurfural (µmol·L^−1^); [-SH]: concentration of total sulfhydryl groups (µmol·g^−1^); [AA]: concentration of ascorbic acid (mg·L^−1^); [Rbf]: concentration of riboflavin (mg·L^−1^); λ_Ftrp_: wavelength of maximum intensity of tryptophan fluorescence; F_Trp_, F_Rbf370_, and F_Rbf450_: maximum fluorescence intensities of tryptophan and riboflavin excited at 370 and 450 nm, respectively.

## Data Availability

The original contributions presented in the study are included in the article; further inquiries can be directed to the corresponding author.
